# Working at Home Through Technology After the Workday Ended: Organizational and Personal Processes Involved and Their Direct and Indirect Effects on Mental Health

**DOI:** 10.5334/pb.1356

**Published:** 2025-04-17

**Authors:** Celestino González-Fernández, Eva Garrosa, Luis Manuel Blanco-Donoso

**Affiliations:** 1 Faculty of Psychology, Autonomous University of Madrid, Madrid, Spain

**Keywords:** technology-assisted supplemental work, workplace flexibility, subjective workplace telepressure, technoaddiction, psychological distress

## Abstract

Workers often make use of Information and Communication Technologies (ICT) in the workplace and outside normal working hours, either voluntarily or compulsorily, especially since the COVID-19 pandemic. This study had three main objectives. Firstly, to explore whether workplace flexibility was associated with technology-assisted supplemental work (TASW), and whether this, in turn, is associated with higher levels of psychological distress. Secondly, to analyze if this relationship between workplace flexibility and TASW could be moderated by subjective workplace telepressure and workers’ technoaddiction. Finally, to investigate whether the execution of this type of supplemental work was linked to psychological distress through the mediating effects of psychological detachment, work-family conflict, and subjective vitality. This cross-sectional study was conducted in 2021 during the pandemic, involved 577 professionals (72.8% women and 27.2% men) from various productive sectors. The participants were primarily from Spain, followed by other Hispanic American countries and European Union countries. Results revealed that workplace flexibility was associated with increased supplemental work, especially among those workers experiencing higher levels of subjective workplace telepressure and technoaddiction. Furthermore, this type of supplemental work was linked to greater psychological distress by hindering psychological disconnection from work, heightening work-family conflict, and reducing feelings of vitality. The discussion has focused on preventive measures.

## Introduction

Technology has blurred the boundaries between the professional and personal/family domains. Individuals can connect from home to carry out work-related tasks, either as a regular mode of work organization (i.e., telecommuting) or to complete unfinished tasks from the workday (i.e., technology-assisted supplemental work; Boswell & Olson-Buchanan, 2007; Fenner & Renn, 2010). Technologies such as smartphones and internet access facilitate the emergence of a reality where workers are constantly available, responding promptly to work demands from any location (Fenner & Renn, 2010; Middleton, 2007). According to the European Working Conditions Survey (Eurofound, 2021), 16% of European workers engage in work-related activities during their leisure time every week to meet work demands.

Remaining constantly connected to work has significant consequences for the personal, family, and organizational dimensions of employees’ lives, impacting their overall quality of life and job satisfaction. Various studies have linked this behavior to elevated levels of anxiety, depression, and stress among workers (Salanova et al., 2007, 2013, 2014; Wang et al., 2008). The pandemic and post-pandemic incidence could have led to increased use of technology outside working hours, influencing the mental health of workers (Aman-Ullah et al., 2023; Tedone, 2022). The magnitude of this issue is underscored by the inclusion of the right to digital disconnection in several European legislations, including the Spanish law (Ley 10/2021, de 9 de julio, de trabajo a distancia; Ley Orgánica 3/2018, de 5 de diciembre, de Protección de Datos Personales y garantía de los derechos digitales). This right has also played a role in shaping organizational policies at the community level (Eurofound, 2021).

**Figure 1:**
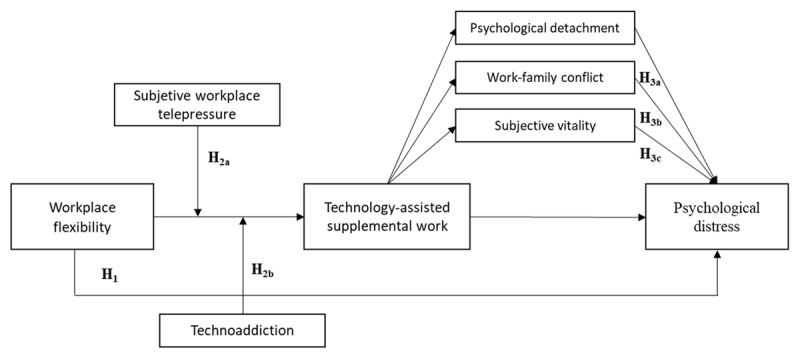
Research model and hypotheses.

In this study, we will address the construct known as Technology-Assisted Supplemental Work (TASW), proposed by Fenner and Renn in 2004, as a phenomenon that represents the lack of disconnection from work during non-working hours. There are workers who, after completing their regular workday, voluntarily reconnect with technology at home to continue working, without any contractual obligation to do so. Given the expected increase in the prevalence of this type of work in the future, there is a growing interest in the scientific community to investigate its antecedents and consequences (Eichberger et al., 2022; Kühner et al., 2023). Specifically, the objectives of this research are as follows: i) To explore whether workplace flexibility is associated with this type of supplementary work outside of regular working hours, and whether this, in turn, is associated with higher levels of psychological distress (i.e., TASW as a potential mediator); ii) to analyze whether the relationship between workplace flexibility and TASW might be moderated by personal characteristics such as subjective workplace telepressure and workers’ technoaddiction; and iii) to examine whether TASW is associated with psychological distress through the mediating effects of psychological detachment, work-family conflict, and subjective vitality (see Figure 1).

### Technology-Assisted Supplemental Work (TASW)

In accordance with Fenner and Renn (2004), TASW refers to the practice of extending working hours and carrying out tasks prescribed by the employer while remaining connected to work, colleagues, supervisors, and other stakeholders of the organization from home (or elsewhere) through Information and Communication Technologies (ICT). This entails activities such as working in the evenings and nights, on weekends, or during vacation periods using work computers and phones (e.g., responding to emails or instant messages, making calls or video conferences, or completing tasks with computers). It is sometimes done to finish unfinished and pending tasks (Eichberger et al., 2022) or due to the inherent nature of the job. Unlike the concept of telecommuting, this is an activity undertaken at the discretion of the employee, after the regular working hours, not covered by any formal agreement between the employee and employer, and for which the latter does not receive compensation (Fenner & Renn, 2010).

Schlachter et al. (2018) proposed an explanatory model regarding the voluntary use of ICT for work outside regular working hours based on a narrative literature review. According to this model, the voluntary use of ICT outside working hours typically translates into an informal extension of working hours when individuals are off-duty, affecting workers’ well-being through the mediation of conflicts between work and the individual’s personal life caused by such usage, as well as the lack of recovery and disconnection. Antecedents for engaging in this type of activity include organizational context factors (i.e., socio-normative context and job characteristics) and individual factors (i.e., preferences, motives, perceptions, and control abilities), as also noted by other authors in literature reviews (i.e., Ďuranová & Ohly, 2016) and meta-analyses (i.e., Kühner et al., 2023). Moreover, within this model, individual variables also serve as moderators for the negative effects of using ICT for work outside regular hours on workers’ well-being (Schlachter et al., 2018). In a recent meta-analysis on technology-assisted work outside regular hours, Kühner et al. (2023) have tested and expanded upon this conceptual model, urging other researchers to continue incorporating specific variables associated with this phenomenon and to investigate processes that could explain its consequences.

### TASW as a Mediator Between Workplace Flexibility and Psychological Distress

Clark (2000) established in his theory of boundaries that each individual plays different roles in the work domain and the personal domain, and that each domain is separated by physical, temporal, and psychological barriers. These boundaries can either reinforce the unique characteristics of each domain or allow a controlled flow between both domains. In fact, boundaries are primarily characterized by their permeability and flexibility.

On one hand, permeability refers to the extent to which the boundary between work and family allows psychological or behavioral aspects of one domain to enter the other (e.g., working from home or addressing family matters at work). On the other hand, flexibility indicates the extent to which a domain can contract or expand to accommodate the demands of another domain (Clark, 2000).

When the boundary between work and family is flexible, individuals tend to have more freedom in choosing their work schedule and location. Thus, the flexibility of boundaries around work can involve aspects such as being able to arrive and leave work at will, having the freedom to work hours that best suit one’s personal agenda, being able to take a day off if needed, or working from a location other than the office. In other words, flexibility alludes to the ability to arrange a work schedule and workspace discretionarily (Clark, 2001, 2002; Hill et al., 2001).

Workplace flexibility is a key condition that allows the crossing of boundaries between work and workers’ personal/family life (Ashforth et al., 2000), and ICT act as tools that create bridges between both domains (Clark, 2000; Tennakoon et al., 2013). From this perspective, flexibility could encourage workers to engage in technology-assisted supplemental work when they are at home after their regular working hours or on non-working days (Tennakoon et al., 2013; Venkatesh & Vitalari, 1992). Even if they have already completed their hours and tasks, the acquired habit of working in flexible hours and locations could lead them to engage in more of this type of work (Ďuranová & Ohly, 2016). Moreover, in general, TASW has been associated with more negative than positive consequences for mental health (González-Fernández et al., 2024). These include reduced sleep duration and quality, difficulties in psychologically disconnecting from work, emotional distress, burnout, job dissatisfaction, and conflicts between work and family (Ďuranová & Ohly, 2016). Taking all this into account, we hypothesize that:

*Hypothesis 1: Workplace flexibility will be directly and significantly associated with technology-assisted supplementary work outside of regular working hours, which in turn will be associated with higher psychological distress, with this type of supplementary work acting as a mediating variable*.

### Telepressure and Technoaddiction in the Flexibility-TASW Relationship

Many workers feel the urgency to respond immediately to calls and emails from their workplaces, often continuously checking their electronic devices, even during non-working hours (Santuzzi & Barber, 2018). This phenomenon is known as subjective workplace telepressure, referring to a psychological state, occurring in response to work-related ICT demands, that consists of (a) perceived preoccupations with work-related ICTs and (b) urges to respond immediately to work-related ICT messages during work and off-work times (Barber & Santuzzi, 2015). This may occur because workers consider it important, as part of their role or with the expectation of gaining benefits, to respond quickly to work demands from their jobs. From this perspective, it is highly likely that workers experiencing this pressure, regardless of the reasons, may connect or make greater use of technology for work purposes outside of working hours (Cambier et al., 2019; Hu et al., 2019).

Additionally, addiction to technologies could lead to supplemental work at home (Turel et al., 2011; Vayre & Vonthron, 2019). Technoaddiction has been defined *“as a specific technostress experience due to an uncontrollable compulsion to use technology ‘everywhere and anytime’ and to use them for long periods of time in an excessive way”* (Salanova et al., 2007, p. 2). Individuals experiencing this type of dependence are psychologically reliant on technology and exhibit discomfort when they don’t have their mobile phones nearby, can’t check their emails throughout the day, or lack access to the Internet or their social networks. Moreover, technoaddiction among workers is often associated with work addiction. In other words, working excessively and compulsively may be linked to technology abuse, especially when technology is commonly used as a work tool (Porter & Kakabadse 2006).

Both subjective workplace telepressure and technoaddiction could reflect failures in the control systems, self-discipline, and self-regulation of workers in using technologies for work purposes outside of working hours, leading to increased supplemental work (Ďuranová & Ohly, 2016; Hu et al., 2019; Turel et al., 2011; Vayre & Vonthron, 2019). Moreover, we suspect that in contexts of high workplace flexibility, telepressure and technoaddiction will enhance the likelihood of technology-assisted supplemental work at home. Having developed the habit of working when and from wherever desired due to the flexibility the position allows, combined with these two personal characteristics, will encourage and reinforce the habit of connecting from home, even when it is not formally or contractually necessary. Considering all these arguments, we hypothesize that:

*Hypothesis 2a) Subjetive workplace telepressure moderate the direct association between workplace flexibility and TASW, so that the association will be stronger for workers with higher levels of subjective workplace telepressure. Hypothesis 2b) Technoaddiction moderate the direct association between workplace flexibility and TASW, so that the association will be stronger for workers with higher levels of technoaddiction.”*

### Consequences of TASW on Worker’s Mental Health: The Mediating Effects of Psychological Detachment, Work-family Conflict and Subjective Vitality

The use of technology at home to fulfill work demands has been linked to a decreased psychological detachment from work (Park et al., 2011) and can serve as a mediator between this type of work and psychological distress (Eichberger et al., 2021). Psychological detachment, in this context, involves not engaging in work tasks or receiving work-related calls after our workday. It implies a disconnection, not only physically but also mentally, from our work (Sonnentag & Fritz, 2007). When workers do not psychologically distance themselves from work, symptoms of depression and fatigue can emerge, among other issues (Sonnentag & Bayer, 2005; Sonnentag & Fritz, 2007). This can be explained by the effort-recovery model (Meijman & Mulder, 1998). The main premise of this model is that when workers do not sufficiently recover from daily efforts at work, their psychophysiological systems continue to operate, leading to high costs in terms of physical and mental health (Sonnentag, 2001). For instance, workers who receive and/or respond to calls and emails outside of working hours or regularly complete unfinished tasks beyond this time may be constantly activating their psychophysiological systems, compromising their physical and mental health. By not allowing their systems to recover from the efforts made during the workday, wear and fatigue responses are likely to appear (Kao et al., 2020).

On the other hand, when workers have to complete work tasks outside of regular working hours, they are extending their commitment to their work role, which can interfere with their commitment to their family role and personal life in terms of time and energy devoted, generating conflicts (Boswell & Olson-Buchanan, 2007; Greenhaus & Beutell, 1985). Our resources are limited, and if we allocate too many resources to one role/domain, the other role will be adversely affected (Edwards & Rothbard, 2000; Kao et al., 2020). When working from home with ICT, one may not be available for the needs of their family or friends, potentially failing to meet their expectations. Constant interruptions from family members can also create confusion about which role is being performed at any given moment (Ashforth et al., 2000). This can lead to conflicts and discomfort. For instance, in the study conducted by Dettmers (2017), it was found that workers who make themselves accessible and available to respond to work demands, formally or informally, outside of working hours (i.e., availability expectations) are the ones experiencing higher emotional exhaustion. This is because their psychological detachment is impeded, leading to increased work-family conflict (Wang et al., 2017). Considering all these arguments, we hypothesize that:

*Hypothesis 3. The relationship between technology-assisted supplemental work and psychological distress will be mediated by 3a) psychological detachment, 3b) work-family conflict and 3c) subjective vitality. Specifically, it is hypothesized that technology-assisted supplemental work will be associated with reduced psychological detachment from work, increased conflict between work and family, and a lower subjective sense of vitality, all of which would be associated with greater psychological distress* (see Figure 1).

## Method

### Sample and Procedure

Firstly, the research received approval from the Research Ethics Committee of the Autonomous University of Madrid, to which the authors of this study are affiliated. Subsequently, participants were contacted using the snowball sampling technique, leveraging the professional networks of the researchers. The inclusion criterion was being a worker over 18 years of age. Those interested in the study were sent a web link containing instructions, informed consent, and a battery of questionnaires developed with Qualtrics software. Participation was voluntary and anonymous. The sample consisted of 577 workers from various professional sectors (450 from Spain, 105 from Hispanic American countries and 22 from the European Union). The socio-professional characteristics of the sample, differentiated by countries and as a whole, can be observed in Table 1.

**Table 1:** Socio-Professional Characteristics of the Sample and Subsamples by Countries.


	SPAIN (*n* = 450)		HISPANIC AMERICAN COUNTRIES (*n* = 105)		EUROPEAN UNION COUNTRIES (*n* = 22)		TOTAL (*n*= 577)	
			
CATEGORICAL VARIABLES	*n*	%	*n*	%	*n*	%	*n*	%

Gender								

*Male*	117	26	33	31,4	7	31,8	157	27,2

*Female*	333	74	72	68,6	15	68,2	420	72,8

Age								

*18 to 29 years*	93	20,7	50	47,6	5	22,7	148	25,6

*30 to 39 years*	106	23,6	22	21	7	31,8	135	23,4

*40 to 49 years*	133	29,6	16	15,2	5	22,7	154	26,7

*50 to 59 years*	93	20,7	11	10,5	5	22,7	109	18,9

*60 to 65 years*	20	4,4	4	3,8	0	0	24	4,2

*More than 65 years*	5	1,1	2	1,9	0	0	7	1,2

Cohabitation Status								

*With Relatives (without Partner or Children)*	68	15,1	34	32,4	2	9,1	104	18

*With Partner*	136	30,2	30	28,6	11	50	177	30,7

*With Partner and Children*	131	29,1	11	10,5	4	18,2	146	25,3

*With Partner, Children, and/or Other Relatives*	14	3,1	0	0	0	0	14	2,4

*Only with Children*	31	6,9	5	4,8	1	4,5	37	6,4

*Alone*	70	15,6	25	23,8	4	18,2	99	17,2

*Missing Values*	0	0,2	0	0	0	0	0	0,2

Children								

*1 Child*	78	17,3	15	14,3	4	18,2	97	16,8

*2 Children*	94	20,9	6	5,7	1	4,5	101	17,5

*3 Children*	19	4,2	5	4,8	2	9,1	26	4,5

*More tan 3 Children*	3	0,7	2	1,9	0	0	5	0,9

*Without Children*	256	56,9	77	73,3	15	6,2	348	60,3

Educational Level								

*No Formal Education*	1	0,2	1	1	0	0	2	0,3

*Certificate of Schooling*	0	0	0	0	1	4,5	1	0,2

*Elementary/Secondary Education*	5	1,1	1	1	0	0	6	1

*High School*	24	5,3	14	13,3	2	9,1	40	6,9

*Bachelor’s/Degree*	128	28,4	61	58,1	5	22,7	194	33,6

*Master’s Degree*	256	56,9	20	19	10	45,5	286	49,6

*Doctorate*	36	8	8	7,6	4	18,2	48	8,3

Current Profession/Occupation								

*Administration and Finance*	45	10	11	10,5	3	13,6	59	10,2

*Research, Development, and Innovation*	36	8	13	12,4	1	4,5	50	8,7

*Educational, Cultural, and Arts Sector*	76	16,9	11	10,5	4	18,2	91	15,8

*Healthcare Sector*	141	31,3	41	39	8	36,4	190	32,9

*Secondary Sector: Industrial, Energy, Mining, and Construction*	17	3,8	1	1	1	4,5	19	3,3

*Social Sector, NGOs, or Non-Profit*	49	10,9	1	1	2	9,1	52	9

*Transportation, Communications, Commercial, Tourism, or Hospitality*	29	6,4	4	3,8	0	0	33	5,7

*Others*	57	12,7	23	21,9	3	13,6	83	14,4

Type of Contract or Employment Sector								

*Internships*	15	3,3	5	4,8	0	0	20	3,5

*Entrepreneur*	16	3,6	3	2,9	0	0	19	3,3

*Public Servant*	32	7,1	2	1,9	0	0	34	5,9

*Self-Employed Worker*	115	25,6	45	42,9	2	9,1	162	28,1

*Temporary Worker*	54	12	7	6,7	7	31,8	68	11,8

*Permanent Worker*	191	42,4	30	28,6	12	54,5	233	40,4

*Others*	27	6	13	12,4	1	4,5	41	7

Do you telecommute?								

*Yes*	312	69,3	82	78,1	13	59,1	407	70,5

*No*	138	30,7	23	21,9	9	40,9	170	29,5

If you telecommute, how many days per week?								

*1 or 2 days*	114	25,3	15	14,3	2	9,1	131	22,7

*3 or 4 days*	68	15,1	27	25,7	4	18,2	99	17,2

*5 or more days*	130	28,9	40	38,1	7	31,8	177	30,7

*Missing Values*	138	30,7	23	21,9	9	40,9	170	29,5


### Variables and Instruments

#### Socio-Professional Data Questionnaire

It includes questions about gender, age, cohabitation status, children, educational level, profession/professional sector, type of contract, telecommuting, and its frequency.

#### Workplace Flexibility

The two subscales of the questionnaire developed by Clark (2002) were employed. The permeability subscale consists of 6 items (e.g., “My family or friends contact me while I am at work”). The flexibility subscale consists of 4 items (e.g., “I can come and go from work when I want”). In both subscales, respondents are required to answer on a Likert-type scale where 1 = never and 5 = always. The scale used has suggested adequate internal consistency in each subscale in validation studies (Clark, 2002) with α = 0.80 for permeability and α = 0.70 for flexibility.

#### Subjective Workplace Telepressure

The questionnaire developed by Barber and Santuzzi (2015) was utilized. It consists of 6 items (e.g., “I feel a strong need to respond to others immediately”) to be answered on a Likert-type scale where 1 = totally disagree and 5 = totally agree. The scale used has shown adequate internal consistency in validation studies (Barber & Santuzzi, 2015) with α = 0.90.

#### Technoaddiction

The subscale from the RED Questionnaire – Technostress (Llorens et al., 2011) was used to measure technoaddiction with 6 items (e.g., “I believe I use technology excessively in my life”). Respondents rate on a Likert-type scale where 0 = never and 6 = always. The subscale used has suggested adequate internal consistency in validation studies (Sánchez-Gómez et al., 2020) with α = 0.92.

#### Technology-Assisted Supplemental Work

The questionnaire developed by Fenner and Renn (2010) was used, comprising five items (e.g., “When I fall behind in my work during the day, I work hard at home at night or on weekends to get caught up by using my cell phone”). Respondents answer on a Likert-type scale where 1 = never and 5 = always. The scale used has shown adequate internal consistency in validation studies (Fenner & Renn, 2010) with α = 0.88.

#### Subjective Vitality

The scale developed by Ryan and Frederick (1997) was used, consisting of seven items (e.g., “I feel alive and full of vitality”). Respondents answer on a Likert-type scale where 1 = not at all and 7 = completely. The scale used has suggested adequate internal consistency in validation studies (Moutão et al., 2013; Rodríguez-Carvajal et al., 2010) with α = 0.91.

#### Work-Family Conflict

The Negative Work-Family Interaction subscale from the SWING Questionnaire developed by Geurts et al. (2005) was used, comprising eight items (e.g., “You are irritable at home because your work is very exhausting”). Respondents rate on a Likert-type scale where 0 = never and 3 = always. The scale used has shown adequate internal consistency in validation studies (Moreno-Jiménez et al., 2009) with α = between 0.77 and 0.89.

#### Psychological Detachment

The Recovery Experience Questionnaire was administered to a Spanish sample (Sanz-Vergel et al., 2010) was used. It includes three items from the psychological detachment subscale (e.g., “After work, I am able to disconnect.”) that are rated on a Likert-type scale ranging from 1 = totally disagree to 5 = totally agree. The subscale used has demonstrated adequate internal consistency in validation studies (Sanz-Vergel et al., 2010) with α = 0.82.

#### Psychological Distress

The DASS-21 Questionnaire (Bados et al., 2005) was used, containing three subscales to assess stress, anxiety, and depression symptoms. It consists of a total of 21 items: anxiety (e.g., “I noticed I had a dry mouth”), depression (e.g., “I couldn’t feel any positive feelings”), stress (e.g., “It was hard for me to relax”). Respondents answer on a Likert-type scale where 0 = did not apply to me and 3 = applied to me a lot or most of the time. In this research, we calculated a global index of psychological distress through the mean of the 21 items. This global measure has proven reliable in validation studies (Fonseca-Pedrero et al., 2010) with adequate internal consistency (α = 0.90).

### Analysis Strategy

All analyses were conducted using IBM SPSS 28.0. The moderation and mediation analyses were carried out utilizing the PROCESS macro v.4.3 developed by Hayes (2017). Specifically, to examine the moderating impact of subjective workplace telepressure and technoaddiction on the relationship between technology-assisted supplemental work and psychological distress, two separate moderation analyses were conducted using PROCESS Model 1, one for each moderator. To investigate the mediating role of TASW in the relationship between workplace flexibility and psychological distress on the one hand, and the mediating role of subjective vitality, work-family conflict, and psychological detachment in the relationship between TASW and psychological distress on the other, PROCESS Model 4 was employed.

To control for the potential effect of sociodemographic variables and given their categorical nature, independent samples t-tests and one-way ANOVA were first conducted to assess whether there were differences based on the different levels of these variables with respect to the central phenomenon of this study, technology-assisted supplementary work, and thus allow their inclusion as covariates in the analysis. The results indicated that there were no differences based on sex (*t* = 1.41; *p* > .05), age (*F* = 2.02, *p* > .05), number of children (*F* = 1.59, *p* > .05), cohabitation status (*F* = 1.38, *p* > .05), level of education (*F* = .922, *p* > .05), nor whether remote working was performed (*F* = 2.07, *p* > .05). However, differences were observed in relation to profession (*F* = 6.18, *p* < .001) and type of contract (*F* = 3.57, *p* > .01), which led us to control for these two variables in the regression models.

The convergent validity of each construct was measured by Average Variance Extracted (AVE), and its value should be above 0.5 (Fornell & Larcker, 1981). In addition, discriminant validity indicates the extent to which a given construct is different from other constructs. Discriminant validity of the measurement model was examined through Fornell and Larcker’s (1981) AVE test and correlations criterion. This test designates that the square root of the respective AVE of each construct should exceed the correlations between the factors making each pair.

## Results

### Descriptive Statistics, Correlations, and Internal Consistency Indices

In Table 2, the means, standard deviations, and internal consistency indices (i.e., Cronbach’s alpha values) of the analyzed variables are presented, along with the correlations among them. Cronbach’s alpha indices indicate desirable reliability for all scales, as they are above .80 and do not exceed the value of .90 (Nunnally & Bernstein, 1994). The perception of workplace flexibility was associated with higher technology-assisted supplemental work, and this supplemental work was correlated with greater psychological distress. Additionally, technology-assisted supplemental work was linked to lower subjective vitality, psychological detachment from work, and increased work-family conflict. Both vitality and detachment were inversely associated with psychological distress, while work-family conflict had a direct association with distress. Thus, all correlations behaved as expected.

**Table 2:** Means, Bivariate Correlations and Cronbach’s alpha (*α*).


	*M*	*SD*	1	2	3	4	5	6	7	8

1. Workplace flexibility	2.95	1.18	(.86)	–.06	–.05	.10*	.11**	–.10*	.17**	–.22**

2. Subjective workplace telepressure	2.94	.92		(.90)	.51**	.26**	–.37**	.31**	–.13**	.37**

3. Technoaddiction	4.16	1.25			(.86)	.44**	–.33**	.47**	–.14**	.38**

4. Technology-assisted supplemental work	2.96	.93				(.82)	–.39**	.48**	–.08*	.25**

5. Psychological detachment	3.16	.95					(.81)	–.46**	.23**	–.35**

6. Work-family conflict	1.94	.58						(.88)	–.35**	.52**

7. Subjective vitality	4.11	1.05							(.84)	–.42**

8. Psychological distress	1.61	.58								(.94)


*Note:* *p < .05; **p < .01; Cronbach’s alpha (*α*) on the diagonal.

### Measurement Model

The convergent validity of the latent variables, assessed via AVE, exceeded 0.5 for most of the variables analyzed (ranged from .46 to .62). Subjective vitality and psychological distress were found to be slightly below this threshold (.46 and .48, respectively). Nevertheless, Fornell and Larcker (1981) asserted that if the AVE is below 0.5 but the composite reliability exceeds 0.6, as was the case in our study (>.70), the construct’s convergent validity can still be deemed adequate. Additionally, the square roots of the average variance extracted (diagonal elements, ranged from .67 to .79) were significantly higher than the correlations between constructs (off-diagonal elements in the corresponding rows and columns, ranged from –.059 to .554). This confirms the discriminant validity of the latent constructs within the model (Barclay et al., 1995). Based on these findings, the measurement model can be considered robust.

### Mediating Effects

#### Workplace Flexibility → TASW → Psychological Distress

**Figure 2:**
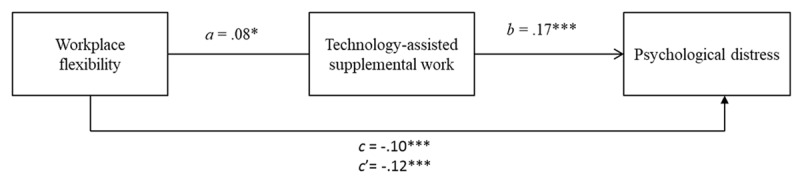
Mediation analysis of technology-assisted supplemental work in the relationship between workplace flexibility and psychological distress. Note: *c* is the total direct effect of the predictive variable on the criterion variable; *c’* is the direct effect of the predictive variable on the criterion variable after controlling for the mediator. *p < .05; ***p < .001.

The variable of workplace flexibility was significantly associated with the mediating variable of TASW. That significant relationship can be observed in Figure 2 through the path *a*. On the other hand, in that figure, we can also observe that TASW was significantly related to the criterion variable (i.e., psychological distress) through path *b*. The total effect of workplace flexibility on psychological distress can be observed in path *c* of Figure 2. This effect was significant. Moreover, the direct effect expressed in path *c*’ was also significant. Indeed, in Table 3, we can confirm that TASW was a significant mediator in the relationship between workplace flexibility and psychological distress. Furthermore, if we observe the change between the total effect (*c*) and the direct effect (*c’*), i.e., when the mediating variables are introduced into the analysis as control variables, the effect of X (i.e., workplace flexibility) on Y (i.e., psychological distress) increases, indicating the existence of a suppression effect. That is, the direct effect (workplace flexibility → psychological distress) is greater than the total effect because the mediator (i.e., TASW) introduces an opposing effect, weakening the overall relationship. Thus, while workplace flexibility is associated with reduced psychological distress, they may have unintended consequences by fostering TASW, thereby exacerbating stress in certain cases. Regarding the effect size, TASW accounted for the explanation of 9% of the total effect (*Pm* = 0.09). Thus, based on these results, we can support hypothesis 1.

**Table 3:** Mediation Analysis.


MODEL PATHWAYS	ESTIMATE	*SE*	95% CI		*Pm*

			LOWER	UPPER	

Total	–.1088	.0200	–.1481	–.0695	

1. Workplace flexibility → Technology-assisted supplemental work → Psychological distress	.0141	.0063	.0020	.0275	0.09

Total	.1602	.0252	.1107	.2096	

1. Technology-assisted supplemental work → Subjective vitality → Psychological distress	.0139	.0076	.0000	.0305	0.08

2. Technology-assisted supplemental work → Work-family conflict → Psychological distress	.1110	.0181	.0077	.0538	0.69

3. Technology-assisted supplemental work → Psychological detachment → Psychological distress	.0295	.0117	.0768	.1476	0.18


*Note: Pm* = ratio between indirect effect and total effect (ab/c). It is an indicator or effect sizes in mediation; CI = Confidence Intervals.

#### TASW → Vitality, Work-family Conflict, and Psychological Detachment → Distress

**Figure 3:**
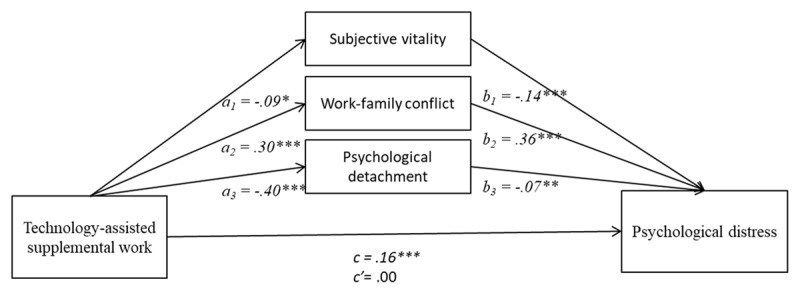
Mediation analysis of subjective vitality, work family-conflict and psychological detachment between technology-assisted supplemental work and psychological distress. Note: *c* is the total direct effect of the predictive variable on the criterion variable; *c’* is the direct effect of the predictive variable on the criterion variable after controlling for the mediator. *p < .05; **p < .01; ***p < .001.

The variable of technology-assisted supplemental work was significantly associated with the mediating variables of subjective vitality, work-family conflict, and psychological detachment. These significant relationships can be observed in Figure 3 through the paths a_1_, a_2_ and a_3_, respectively. On the other hand, in that figure, we can also observe that all three mediators were significantly related to the criterion variable (i.e., psychological distress) through paths b_1_, b_2_, and b_3_ within the figures.

The total effect of technology-assisted supplemental work on psychological distress can be observed in path c of Figure 3. This effect was significant. However, the direct effect expressed in path c’ was not significant, which raises suspicion about the presence of an indirect effect through the three mediating variables. Indeed, in Table 3, we can confirm that subjective vitality, work-family conflict, and psychological detachment were three significant mediators in the relationship between technology-assisted supplemental work and psychological distress. Furthermore, if we observe the change between the total effect (*c*) and the direct effect (*c’*), i.e., when the mediating variables are introduced into the analysis as control variables, the effect of X (i.e., technology-assisted supplemental work) on Y (i.e., psychological distress) disappears, indicating the existence of full mediation. Regarding the effect size, work-family conflict is the variable with the greatest effect if we consider the value of the estimates, accounting for the explanation of 69% of the total effect (*Pm* = 0.69), compared to 18% and 8% for psychological detachment and subjective vitality, respectively. Thus, based on these results, we can support hypothesis 3a, 3b and 3c.

### Moderator Effects

In Table 4, we can observe the moderation analysis for the variable of subjective workplace telepressure in the direct relationship between workplace flexibility and technology-assisted supplemental work. As seen in the table, the model was significant, and both workplace flexibility and subjective workplace telepressure found significant direct effects on the criterion variable. Additionally, there was a significant interaction between both variables, indicating the hypothesized moderation effect in hypothesis 2a.

**Table 4:** Moderation Analysis for the Variable of Subjective Workplace Telepressure.


	TECHNOLOGY-ASSISTED SUPLEMENTAL WORK					

	*β*	*SE*	*t*	*p*	CONFIDENCE INTERVALS	

					LOWER	UPPER

Constant	2.56	.48	5.29	.000	1.6156	3.5190

Type of Contract or Employment	.01	.02	.89	.372	–.0217	.0579

Current Profession/Occupation	.01	.01	.69	.486	–.0199	.0418

Workplace flexibility	.08	.03	2.57	.010	.0197	.1472

Subjetive workplace telepressure	.26	.04	6.57	.000	.1859	.3443

Workplace flexibility × S. Workplace Telepressure	.08	.03	2.54	.011	.0190	.1466

		*R*	*R2*	*F*	*p*	

		.3105	.0964	12.18	.000	


**Figure 4:**
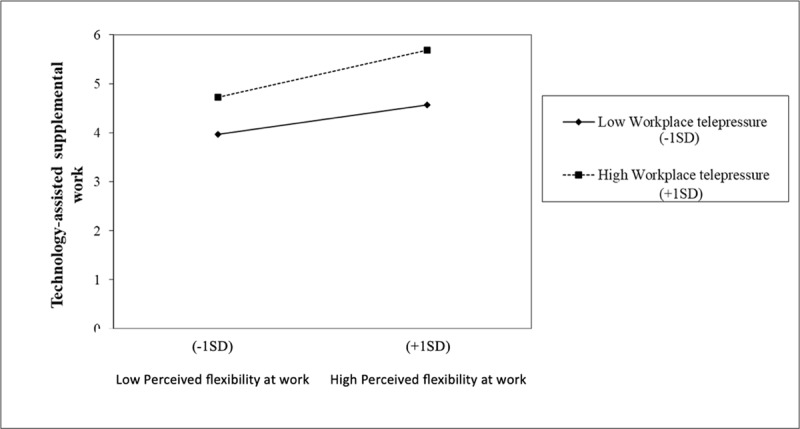
Moderating effect of subjective workplace telepressure between perceived workplace flexibility and technology-assisted supplemental work.

Figure 4 illustrates this moderation effect. As shown in it, the relationship between workplace flexibility and supplemental work is always direct. Specifically, the slope was significant both for individuals exhibiting higher levels of subjective workplace telepressure (*γ* = 0.404; *p* < .01; *t =* 3.04, *p* < 01), and for those with low levels of subjective workplace telepressure (*γ* = 0.251; *p* < .01; *t =* 3.35, *p* < 01). However, the slope coefficient was higher in the case of workers with high levels of subjective workplace telepressure. That is to say, as established in hypotheses 2a, it is those workers who perceive greater flexibility between the work and personal domains who extend their workday at home the most through technology, especially among those who feel a high pressure to meet the technological demands they face.

In Table 5, we can see the moderation analysis for the variable of technoaddiction in the existing direct relationship between workplace flexibility and technology-assisted supplemental work. As observed in the table, the model was significant, and both workplace flexibility and technoaddiction showed significant direct effects on the criterion variable. Additionally, there was a significant interaction between them, showing the hypothesized moderation effect in hypothesis 2b.

**Table 5:** Moderation Analysis for the Variable of Technoaddiction.


	TECHNOLOGY-ASSISTED SUPLEMENTAL WORK					

	*β*	*SE*	*t*	*p*	CONFIDENCE INTERVALS	

					LOWER	UPPER

Constant	2.94	.45	6.52	.000	2.0548	3.8261

Type of Contract or Employment	.03	.01	1.63	.103	–.0063	.0677

Current Profession/Occupation	–.00	.01	–.19	.843	–.0316	.0258

Workplace flexibility	.08	.03	2.92	.003	.0288	.1470

Technoaddiction	.33	.02	12.16	.000	.2805	.3885

Workplace flexibility × Technoaddiction	.04	.02	2.02	.043	.0013	.0886

		*R*	*R2*	*F*	*p*	

		.4721	.2229	32.75	.000	


**Figure 5:**
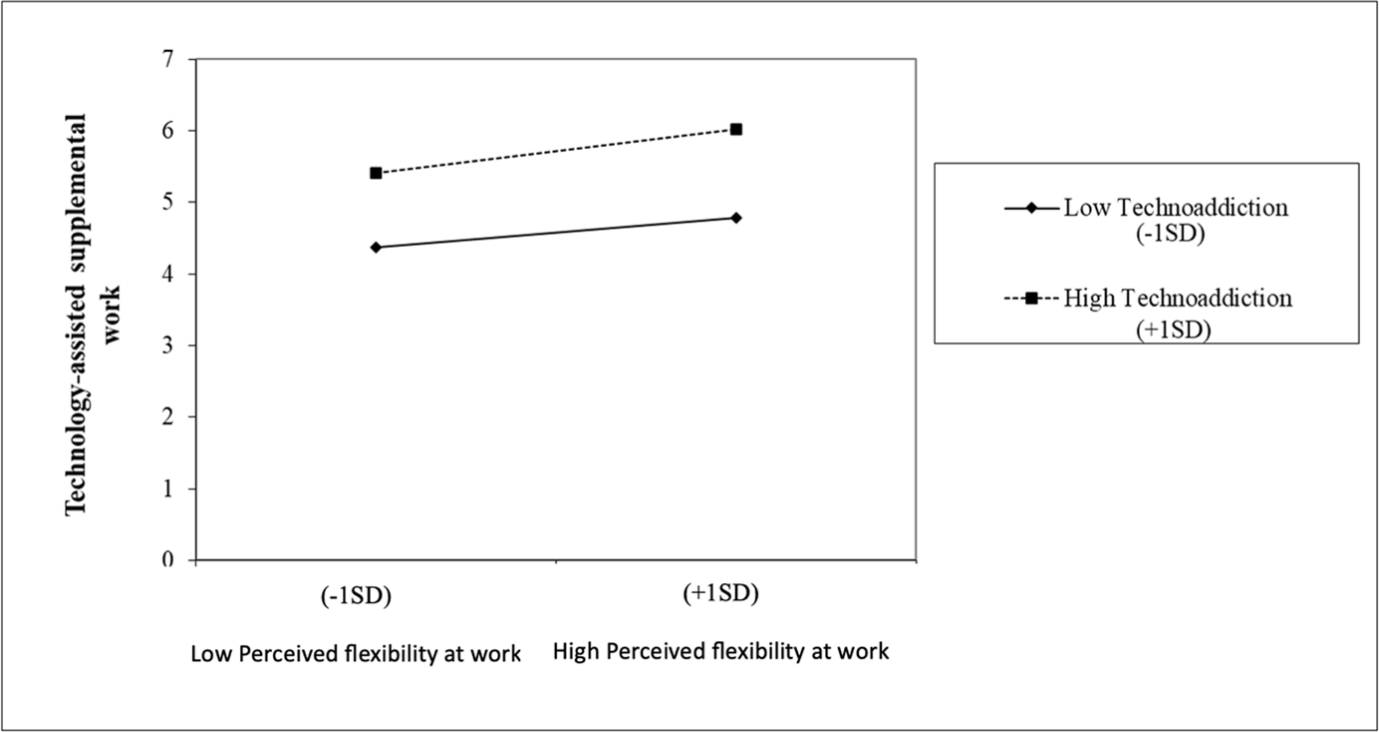
Moderating effect of technoaddiction between perceived workplace flexibility and technology-assisted supplemental work.

Figure 5 illustrates this moderation effect. As seen in it, the relationship between perceived workplace flexibility and supplemental work is always direct. Specifically, the slope was significant both for individuals exhibiting higher levels of technoaddiction (*γ* = 0.331; *p* < .01; *t =* 2.65, *p* < 01), and for those with low levels of techoaddiction (*γ* = 0.218; *p* < .01; *t =* 3.05, *p* < 01). However, the slope coefficient was higher in the case of workers with high levels of technoaddiction. That is to say, as established in hypotheses 2b, it is those workers who perceive greater flexibility between the work and personal domains and those who exhibit higher levels of technoaddiction who extend their workday at home the most through technology.

## Discussion

The main aim of this research was to investigate whether workplace flexibility was associated with greater participation in technology-assisted supplemental work outside regular working hours and whether this, in turn, increased workers’ psychological distress levels. Additionally, we examined whether the relationship between workplace flexibility and such supplemental work was moderated by workers’ subjective workplace telepressure and technoaddiction. Finally, we explored whether engaging in this type of supplemental work was linked to heightened psychological distress through the mediating effects of psychological detachment, work-family conflict, and subjective vitality. In light of the results obtained, this study makes a theoretical contribution to the exploration of technology-assisted supplemental work, delving into newly explored antecedents and consequences (Rodríguez-Muñoz, 2019), and to its practical application, aiming to provide preventive solutions and promote health within organizations.

Our results showed, firstly, that workplace flexibility was associated with increased technology-assisted supplemental work in line with previous studies (Clark, 2000; Fenner & Renn, 2004). Workplace flexibility allows for crossing boundaries between work and workers’ personal lives (Ashforth et al., 2000), and ICT acts as tools that create links between both domains (Clark, 2000; Tennakoon et al., 2013), which would encourage workers to engage in technology-assisted supplemental work when they are at home after their regular working hours (Tennakoon et al., 2013; Venkatesh & Vitalari, 1992). As pointed out by Ďuranová and Ohly (2016), the acquired habit of working in flexible hours and locations could lead to more engagement in this type of work. Additionally, engaging in technology-assisted supplemental work outside regular working hours was associated with higher psychological distress. Specifically, the results of the mediation analyses revealed an interesting suppression effect, suggesting that while workplace flexibility is associated with lower psychological distress, it may also have unintended consequences by fostering a culture of constant availability outside regular working hours and promoting technology-assisted supplemental work, thereby exacerbating stress under these circumstances. At a practical level, these findings have potential implications across various levels. At the organisational level, the application of existing laws and regulations governing technology use in the workplace, alongside efforts to address resistance rooted in organisational culture, can help to reduce self-imposed obligations (Eichberger et al., 2022; Kühner et al., 2023). Training, ongoing support, and regular risk assessments are critical to fostering a healthy relationship with technology. Organisations should cultivate a culture that clearly separates professional and personal life, discouraging the extension of working hours through digital devices. Leadership is pivotal: leaders must exemplify healthy behaviours, respect rest periods, and provide employees with clear roles, responsibilities, and adequate resources. Raising awareness of the psychosocial risks associated with ICT use through training and awareness campaigns can assist employees in better managing work-life balance, recovery, and overall well-being. At the individual level, workers can enhance task organisation and time management to avoid overworking at home. Strategies to maintain boundaries between work and personal life, such as rituals that facilitate disconnection, are essential (Rothbard & Ollier-Malarrete, 2015). Awareness of habits related to work and technology use is a key initial step and may necessitate training, awareness-raising initiatives, or professional support provided by organisations. Preventive measures should aim to avoid the misuse of flexible work arrangements to extend working hours unnecessarily (Manzano, 2018).

Secondly, our results also show that technology-assisted supplemental work is associated with higher psychological distress through the mediating effect of subjective vitality, work-family conflict, and psychological detachment (Ďuranová & Ohly, 2016; Eichberger et al., 2022; Kühner et al., 2023; Sonnentag & Bayer, 2005; Sonnentag & Fritz, 2007). The use of technology at home to fulfill work demands has been linked to lower psychological detachment (Park et al., 2011), which results in higher distress (Eichberger et al., 2021; Sonnentag & Bayer, 2005; Sonnentag & Fritz, 2007). As noted by the effort-recovery model (Meijman & Mulder, 1998), if workers do not have sufficient recovery from their daily work, their psychological and physiological systems may remain overactivated when they are at home, negatively impacting their physical and mental health (Sonnentag, 2001). Specifically, one of the most visible effects of the continued effort involved in this type of supplemental work could be a decrease in the levels of vitality experienced by workers, as their energy resources are depleted by engaging in activities other than recovery (Ďuranová & Ohly, 2016; Wang et al., 2017).

Additionally, supplemental work increases work-family and personal life conflicts, which are also associated with higher psychological distress. When working from home with ICT, individuals may not be available for the needs of their family or friends and may fail to meet their expectations. They may also be constantly interrupted by them, leading to feelings of stress, anxiety, and sadness among workers due to the conflict between these domains (Ashforth et al., 2000). At a practical level, these findings highlight the importance of the preventive strategies outlined in the previous paragraph to minimise supplementary work facilitated by technology as much as possible. However, in some cases, it may be necessary and unavoidable for employees to engage in this type of work. To mitigate its consequences, it would be beneficial to implement recovery strategies, such as developing disconnection routines (e.g., walks and leisure activities), practising relaxation exercises and/or meditation (e.g., mindfulness), engaging in reinforcing hobbies and activities, fostering social contact and support, and establishing good sleep habits. Organisations can also contribute by fostering a culture of disconnection and recovery. This may involve promoting well-being and care programmes, encouraging breaks and rest periods, offering flexible working hours and locations, and providing social support to employees when risk situations are identified.

Finally, it has been suggested that the aforementioned effect of workplace flexibility is moderated both by workers’ subjective workplace telepressure and technoaddiction. Specifically, it has been found that the relationship between workplace flexibility and technology-assisted supplemental work is stronger among those workers who exhibit higher subjective workplace telepressure (Cambier et al., 2019; Hu et al., 2019) and greater technoaddiction (Turel et al., 2011; Vayre & Vonthron, 2019). Thus, in contexts of high workplace flexibility, subjective workplace telepressure and technoaddiction are likely to increase the likelihood of technology-assisted supplemental work at home, probably because workplace flexibility allows the development of a habit of working whenever and wherever one wants. Considering that both subjective workplace telepressure and technoaddiction may reflect failures in workers’ control systems, flexibility may lead to increased supplemental work (Ďuranová & Ohly, 2016; Hu et al., 2019; Turel et al., 2011; Vayre & Vonthron, 2019), fostering and reinforcing the habit of connecting from home, even when it is not formally or contractually necessary. While those workers with higher subjective workplace telepressure might do so due to a sense of urgency, need, and concern if they do not respond to work demands (Barber & Santuzzi, 2015), and even because immediately responding to these demands may be a valued behavior for them, those workers with greater technoaddiction might do so under the compulsion and dependence on technology and work that they have difficulty controlling (Porter & Kakabadse 2006; Turel et al., 2011; Vayre & Vonthron, 2019). At a practical level, it is important for workers in highly flexible work environments to learn and implement strategies for technological self-regulation, promoting conscious use of digital tools—such as scheduling regular breaks and limiting time spent interacting with work-related devices, for example, through location-based segmentation techniques. To address work-related telepressure, it may be useful to develop cognitive and behavioural stress management skills, such as mindfulness and conscious breathing, thought restructuring, or assertive behaviour while practising delays in responding to technological work demands. Negotiating expectations regarding response times and availability could also reduce perceptions of telepressure and promote a more balanced work environment. Cultivating intra-work digital disconnection habits, such as incorporating non-technological activities like brief in-person meetings, stretching exercises, or breaks in device-free areas, can help interrupt cycles of hyperconnection and foster healthier interactions with digital tools. The organisation can contribute to many of these issues by designing responsible technology use policies and establishing clear guidelines on the use of digital tools— including response time limits and disconnection rules. This will reduce expectations of constant availability and help alleviate telepressure. It should also provide training for employees, particularly those with high work flexibility, on the efficient and strategic use of technological tools to encourage controlled interaction with devices and prevent compulsive behaviours linked to technology addiction. Finally, the organisation can offer the necessary resources to support employees who engage in a deregulated and negative relationship with technology for work purposes, by providing employee assistance programmes.

An important aspect to highlight and consider when interpreting the results is that the research was conducted in 2021, during the COVID-19 pandemic. During this period, numerous changes occurred in the organisation of work in many job positions. For instance, many workers transitioned to remote working, and working hours became more flexible, leading to a blurring of the boundaries between work and employees’ personal and family lives. Additionally, in many cases, tasks and functions were reorganised, work pace and workloads increased, and multitasking became more prevalent. Individuals experiencing significant personal pressure to meet these demands, coupled with a lack of behavioural regulation as reflected in telepressure and technoaddiction explored in this study, may have found themselves engaging in more technology-assisted work outside of regular working hours. This, in turn, could have adversely affected their mental health by reducing their energy levels, increasing conflicts with family life, and offering fewer opportunities for recovery. Therefore, although these effects were observed during the pandemic, potentially influenced by the circumstances and changes experienced at that time, it would be of great interest for future studies to further investigate the relationships identified in this study once the pandemic has ended.

This study presents some limitations. Firstly, the most relevant one is related to the cross-sectional design of the study. One inherent limitation of cross-sectional studies is their inability to establish causality. In other words, since our data were collected at a single point in time, it is difficult to determine whether the relationship between two variables is causal or merely correlational. This limitation requieres a cautious interpretation of the results, as establishing temporal precedence is essential for causal inference—something that this cross-sectional design cannot provide. For example, we have hypothesised, among other things, that supplementary work affects psychological stress by being more strongly associated with a lack of psychological detachment from work. However, this relationship could also be bidirectional: Individuals who struggle to disconnect from work may end up working beyond their usual hours, thus increasing their psychological stress. Establishing causality is what is not possible with a cross-sectional design. Nevertheless, the fact that the hypotheses of this study are based on sound premises and a robust theoretical model aims to mitigate this limitation. Another issue associated with correlational designs is common method variance. This problem arises when one or more variables are measured using the same method. For example, in our case, responding to a questionnaire on workplace flexibility could influence the answers given to a questionnaire on technology-assisted supplemental work, especially when both are administered at the same time. However, much of the content of the analysed variables is quite distinct, and from this perspective, this bias tends to have a less pronounced effect, although it cannot be completely ruled out. Finally, there was an imbalance in the sample size between workers from Spain (the majority) compared to other countries, which necessitated a combined analysis of the data.

Despite all of this, this research also demonstrates some strengths. The main one being the achievement of a large and significant number of participants, and their diverse professional backgrounds, allowing for better generalization of the results. Furthermore, we believe that the findings of this study provide an original contribution and help to expand the current understanding of new forms of work organisation and their effects on workers’ behaviour and affect.

In future research, it would be appropriate to consider longitudinal studies that facilitate the observation and relationships of variables over time. The continuous technological and social changes make it difficult for explanatory models to remain static, and they need to be updated contingently (Eurofound, 2021). From this perspective, further investigation is needed into the processes preceding and causing increased technology-assisted supplemental work, as well as its consequences, with robust methodological designs (Kühner et al., 2023). It would also be interesting to contribute a qualitative perspective and methodology to these studies, delving into cross-cultural analyses of the variables and their relationships. Investigating whether the relationships between personal and job demands have different weight and effects among countries, and whether they can vary by culture and labor policies in each area, would be truly insightful. Lastly, the issues analyzed in this study and the future research proposed could be enriched by incorporating a gender perspective. In this study, the majority of participants were women (almost three-quarters of the sample), so the demands and consequences associated with the influence of moderating and mediating variables may vary by gender.

## Conclusion

Technology-assisted supplemental work outside of regular working hours has experienced significant growth in recent years, transforming the way people perform their work tasks aided by workplace flexibility, impacting the quality of life and mental health of workers. Experiences such as subjective workplace telepressure or technoaddiction have proven to be relevant demands when understanding the change in this type of work and its detrimental effect on occupational health. Other variables such as subjective vitality, work-family conflict, and psychological detachment have also been explanatory in understanding the detrimental effect of these labor processes that extend beyond the regular workday. It is about implementing preventive and intervention measures to effectively address, assess, and treat these demands and issues within organizations.

## Data Accessibility Statement

The data that support the findings of this study are available from the corresponding author upon reasonable request.
